# [2,2′-Imino­diethano­lato(2−)-κ^3^ *O*,*N*,*O*′][4-(meth­oxy­carbonyl­meth­yl)phen­yl]boron

**DOI:** 10.1107/S1600536810037864

**Published:** 2010-09-30

**Authors:** Ahmed L. Zein, Louise N. Dawe, Paris E. Georghiou

**Affiliations:** a Department of Chemistry, Memorial University of Newfoundland, St Johns, NL, Canada A1B 3X7

## Abstract

The title compound, C_13_H_18_BNO_4_, was readily obtained from the reaction of methyl 4-boronobenzene acetate with ethano­lamine. A combination of inter­molecular N—H⋯O hydrogen bonds and C—H⋯π inter­actions leads to the pairwise association of mol­ecules.

## Related literature

For background to the biological importance of boron, see: Warrington (1923 ▶); Jabbour *et al.* (2004 ▶). For the use of boron-containing reagents in synthetic chemistry, see: Miyaura & Suzuki (1995 ▶); Corey *et al.* (1987 ▶); Liu *et al.* (2007 ▶); Jung & Lazarova (1999 ▶); Chan *et al.* (1998 ▶); Evans *et al.* (1998 ▶); Lam *et al.* (1998 ▶). For related structures, see: Rettig & Trotter (1975 ▶); Wang & Georghiou (2002 ▶).
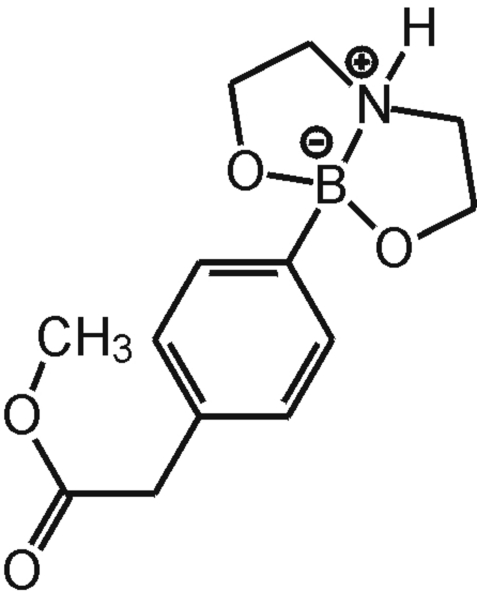


## Experimental

### 

#### Crystal data

C_13_H_18_BNO_4_
*M* *_r_* = 263.10
Orthorhombic, 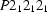

*a* = 8.3776 (11) Å
*b* = 8.9269 (11) Å
*c* = 17.369 (2) Å
*V* = 1299.0 (3) Å^3^
*Z* = 4
Mo *K*α radiation
μ = 0.10 mm^−1^
*T* = 153 K
0.30 × 0.09 × 0.06 mm

#### Data collection

Rigaku Saturn diffractometer
Absorption correction: numerical (*NUMABS*; Higashi, 1999 ▶) *T*_min_ = 0.985, *T*_max_ = 0.997
16363 measured reflections
1725 independent reflections
1707 reflections with *I* > 2σ(*I*)
*R*_int_ = 0.037

#### Refinement

*R*[*F* ^2^ > 2σ(*F* ^2^)] = 0.043
*wR*(*F* ^2^) = 0.112
*S* = 1.17
1725 reflections
173 parameters
H-atom parameters constrained
Δρ_max_ = 0.20 e Å^−3^
Δρ_min_ = −0.22 e Å^−3^

### 

Data collection: *CrystalClear* (Rigaku, 2002 ▶); cell refinement: *CrystalClear*; data reduction: *CrystalClear*; program(s) used to solve structure: *SHELXS97* (Sheldrick, 2008 ▶); program(s) used to refine structure: *SHELXL97* (Sheldrick, 2008 ▶); molecular graphics: *ORTEP-3* (Farrugia, 1997 ▶); software used to prepare material for publication: *publCIF* (Westrip, 2010 ▶).

## Supplementary Material

Crystal structure: contains datablocks I, global. DOI: 10.1107/S1600536810037864/fj2337sup1.cif

Structure factors: contains datablocks I. DOI: 10.1107/S1600536810037864/fj2337Isup2.hkl

Additional supplementary materials:  crystallographic information; 3D view; checkCIF report

## Figures and Tables

**Table 1:** Hydrogen-bond geometry (Å, °) *Cg*3 is the centroid of the C1–C6 ring.

*D*—H⋯*A*	*D*—H	H⋯*A*	*D*⋯*A*	*D*—H⋯*A*
N1—H1⋯O2^i^	0.93	2.06	2.921 (2)	154
C10—H10*B*⋯*Cg*3^ii^	0.99	2.65	3.618 (2)	166

Symmetry codes: (i) 
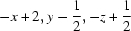
; (ii) 
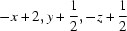
.

**Fig. 1.**
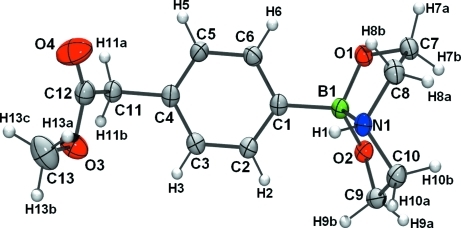
The molecular structure of (2), with atom labels and 50% probability displacement ellipsoids for non-H atoms.

**Fig. 2.**
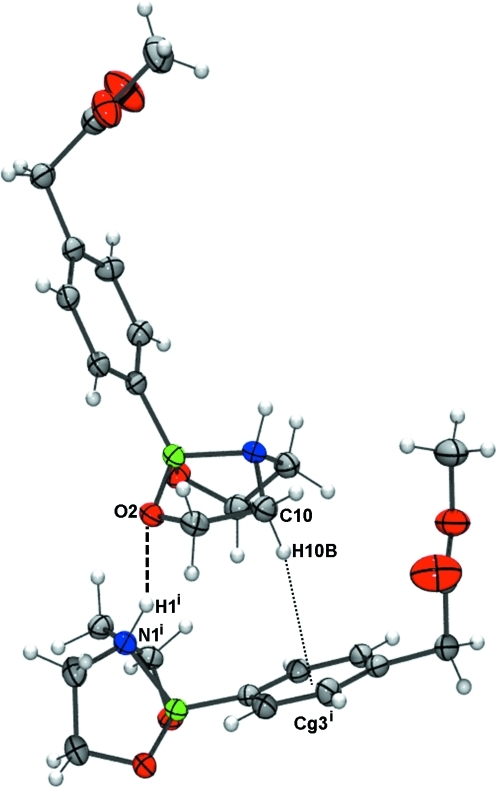
Intermolecular hydrogen bonds (long dashes) and C—H···π interactions (short dashes) between two associated molecules.

**Fig. 3.**
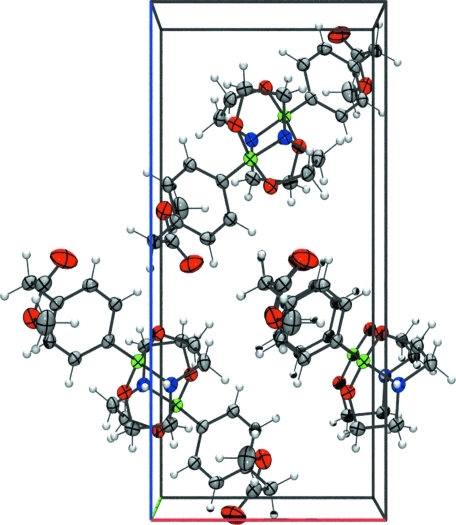
Unit cell viewed perpendicular to the b axis, showing the pair-wise ordering of molecules in the crystal lattice. (Cell axes: a = red, b = green, c = blue)

## supplementary crystallographic information

### Comment

Boron is known to be an important trace element in higher plants (Warrington, 1923) and boron-containing compounds have been shown to have a range of diverse biological acvtivities (Jabbour *et al.*, 2004). The use of boron-containing reagents is also widespread in synthetic chemistry and this is due mainly to the pioneering work of H. C. Brown and coworkers and Suzuki and his coworkers (Miyaura & Suzuki, 1995). Corey, Bakshi and Shibata (Corey *et al.*, 1987) discovered that a chrial oxazaborolidine ("CBS" reagent) which contains both boron-nitrogen and boron-oxygen bonds was capable of effecting enantioselective reduction of prochiral ketones, imines, and oximes to produce chiral alcohols, amines, and amino alcohols in excellent yields and ee's. Corey's group has also shown that chiral oxazaborolidine-aluminium bromide complexes (Liu *et al.* 2007) are also effective catalysts for enantioselective Diels-Alder reactions. In principle, oxazaborolidines are derived from reactions of a boronic acid and aminoalcohols and a less well known application of oxazaborolidines is to facilitate the conversion of a pinacolatoborane, by mild acid-catalysis (Jung & Lazarova, 1999), to the corresponding boronic acid, a key step for cupric acetate promoted coupling of an arylboronic acids with phenols (Chan *et al.*, 1998; Evans *et al.*, 1998; Lam *et al.* 1998).

There has been only one reported X-ray crystallographic study of the structure of a diethanolamine ester of a phenylboronic acid (1) (Rettig & Trotter, 1975). This compound which was named as *B*-phenyl-*diptych*boroxazolidine (alternative names: Tetrahydro-[1,3,2]oxaza-borol[2,3-*b*][1,3,2]oxazaborole; [[2,2'-(Imino-kN)bis[ethanolato-kO]](2-)]phenylboron) was measured on a diffractometer with Cu *K_a_* radiation and it was revealed to be non-centrosymmetric and in the *P*2_1_ space group. The absolute configuration of the enatiomorphic crystal was determined in this study. In connection with our own work (Wang & Georghiou, 2002), crystals of (2), the corresponding diethanolamine ester of the 4-boronic acid derivative of methyl phenylacetate, were obtained and the structure of the molecule is reported here.

Methyl *p-*[[2,2'-iminobis[ethanolato]](2-)-*N*,*O*,*O*']phenylacetateboron (2; Figure 1) crystallized in the non-centrosymmetric space group *P*2_1_2_1_2_1_, however, data collection was performed using molybdenum radiation, and the absolute configuration could not be determined due to the lack of an atom with significant anomalous dispersion. Intermolecular hydrogen bonding between N1—H1···O2^i^ (N1···O2^i^ = 2.921 (2) Å) and C—H···π interactions between C10—H10B···*Cg*3^ii^ (C10···*Cg*3^ii^= 3.618 (2); where *Cg*3 is the centroid of C1—C6) leads to the pair-wise association of molecules (Figure 2). These molecular associates are related *via* the twofold screw axes in the crystal structure (viewed perpendicular to the *b* axis in Figure 3).

### Experimental

To a solution of PdCl_2_(dppf) (160 mg, 0.18 mmol) in dioxane (24 ml) was added methyl 4-(trifluoroacetoxyacetate (1.58 g, 5.93 mmol), Et_3_N (2.49 ml, 17.8 mmol) and 4,4,5,5-tetramethyl-1,3,2-dioxaborolane (1.30 ml, 8.9 mmol). After stirring for 20 h at 100 *^o^*C, the reaction mixture was extracted with benzene. The extract was purified by flash column chromatography (silica gel, 10%EtOAc in hexanes) to afford the arylboronate (1.47 g, 90%) ^1^HNMR (500 MHz, CDCl_3_) 1.34 (s, 12H), 3.64 (s, 2H), 3.67(s, 3H), 7.27(d, *J*=10 Hz, 2H), 7.77(d, *J*=10 Hz, 2H). To a solution of the arylboronate (1.47 g, 5.3 mmol) in diethylether (53 ml) was added diethanolamine (0.6 ml, 5.8 mmol) in 2-propanol (10 ml). The resulting mixture was stirred at ambient temperature for 72 h, the reaction mixture was then filtered and the solid was washed with diethyl ether to give cyclic aminoarylboronate (1.08 g, 77%) as a colorless powder. Crystals suitable for X-ray diffraction analysis were obtained by crystallization from ethyl acetate solution. ^1^H NMR (500 MHz, CDCl_3_) 2.49–2.51(m, 2H), 2.96–3.03(m, 2H), 3.58(s, 2H), 3.63(s, 3H), 3.70–3.72(m, 2H), 3.79–3.74(m, 2H), 5.83(s, 1H), 7.14(d, *J*=7.5 Hz, 2H), 7.43(d, *J*=7.5 Hz, 2H); ^13^C NMR 41.0, 51.1, 51.8, 63.2, 128.3, 132.7, 132.8, 172.6.

### Refinement

H atoms were found in difference Fourier maps and subsequently placed in idealized positions with constrained distances and with *U*_iso_(H) values set to either 1.2Ueq or 1.5Ueq of the attached atom. They were refined on a riding model. All non-hydrogen atoms were refined anisotropically. This crystal was a weak anomalous scatterer collected with MoKa radiation, therefore, Friedel mates were merged (MERG 4) and absolute configuration was not determined.

### Crystal data

**Table:**

C_13_H_18_BNO_4_	*D*_x_ = 1.345 Mg m^−^^3^
*M**_r_* = 263.10	Melting point = 452–453 K
Orthorhombic, *P*2_1_2_1_2_1_	Mo *K*α radiation, λ = 0.71075 Å
Hall symbol: P 2ac 2ab	Cell parameters from 5108 reflections
*a* = 8.3776 (11) Å	θ = 2.6–30.6°
*b* = 8.9269 (11) Å	µ = 0.10 mm^−^^1^
*c* = 17.369 (2) Å	*T* = 153 K
*V* = 1299.0 (3) Å^3^	Platelet, colorless
*Z* = 4	0.30 × 0.09 × 0.06 mm
*F*(000) = 560	

### Data collection

**Table:**

Rigaku Saturn diffractometer	1725 independent reflections
Radiation source: fine-focus sealed tube	1707 reflections with *I* > 2σ(*I*)
graphite - Rigaku SHINE	*R*_int_ = 0.037
Detector resolution: 14.63 pixels mm^-1^	θ_max_ = 27.5°, θ_min_ = 2.6°
ω scans	*h* = −10→10
Absorption correction: numerical (*NUMABS*; Higashi, 1999)	*k* = −11→11
*T*_min_ = 0.985, *T*_max_ = 0.997	*l* = −22→22
16363 measured reflections	

### Refinement

**Table:**

Refinement on *F*^2^	Primary atom site location: structure-invariant direct methods
Least-squares matrix: full	Secondary atom site location: difference Fourier map
*R*[*F*^2^ > 2σ(*F*^2^)] = 0.043	Hydrogen site location: inferred from neighbouring sites
*wR*(*F*^2^) = 0.112	H-atom parameters constrained
*S* = 1.17	*w* = 1/[σ^2^(*F*_o_^2^) + (0.0529*P*)^2^ + 0.4126*P*] where *P* = (*F*_o_^2^ + 2*F*_c_^2^)/3
1725 reflections	(Δ/σ)_max_ < 0.001
173 parameters	Δρ_max_ = 0.20 e Å^−^^3^
0 restraints	Δρ_min_ = −0.22 e Å^−^^3^

### Special details

**Table:**

Geometry. All e.s.d.'s (except the e.s.d. in the dihedral angle between two l.s. planes) are estimated using the full covariance matrix. The cell e.s.d.'s are taken into account individually in the estimation of e.s.d.'s in distances, angles and torsion angles; correlations between e.s.d.'s in cell parameters are only used when they are defined by crystal symmetry. An approximate (isotropic) treatment of cell e.s.d.'s is used for estimating e.s.d.'s involving l.s. planes.
Refinement. Refinement of *F*^2^ against ALL reflections. The weighted *R*-factor *wR* and goodness of fit *S* are based on *F*^2^, conventional *R*-factors *R* are based on *F*, with *F* set to zero for negative *F*^2^. The threshold expression of *F*^2^ > σ(*F*^2^) is used only for calculating *R*-factors(gt) *etc*. and is not relevant to the choice of reflections for refinement. *R*-factors based on *F*^2^ are statistically about twice as large as those based on *F*, and *R*- factors based on ALL data will be even larger.The crystal was a weak anomalous scatterer collected with Mo *K*α radiation. Friedel mates were merged (MERG 4) and the absolute configuration was not determined.

### Fractional atomic coordinates and isotropic or equivalent isotropic displacement parameters (Å^2^)

**Table:**

	*x*	*y*	*z*	*U*_iso_*/*U*_eq_	
O1	1.00908 (19)	0.47529 (18)	0.35582 (9)	0.0281 (4)	
O2	0.85897 (19)	0.47478 (17)	0.23631 (9)	0.0268 (4)	
O3	0.5232 (2)	−0.2735 (2)	0.39057 (11)	0.0422 (5)	
O4	0.6510 (3)	−0.2494 (3)	0.50205 (13)	0.0617 (7)	
N1	1.0730 (2)	0.2952 (2)	0.25504 (10)	0.0249 (4)	
H1	1.0599	0.1919	0.2577	0.030*	
C1	0.7998 (3)	0.2734 (2)	0.34181 (12)	0.0243 (4)	
C2	0.6576 (3)	0.2247 (3)	0.30806 (13)	0.0253 (4)	
H2	0.6285	0.2624	0.2589	0.030*	
C3	0.5568 (3)	0.1220 (3)	0.34475 (13)	0.0261 (5)	
H3	0.4626	0.0888	0.3195	0.031*	
C4	0.5932 (3)	0.0679 (3)	0.41775 (13)	0.0263 (5)	
C5	0.7326 (3)	0.1174 (3)	0.45272 (13)	0.0296 (5)	
H5	0.7589	0.0827	0.5028	0.035*	
C6	0.8342 (3)	0.2173 (3)	0.41551 (13)	0.0276 (5)	
H6	0.9294	0.2482	0.4406	0.033*	
C7	1.1685 (3)	0.4963 (3)	0.33084 (14)	0.0302 (5)	
H7A	1.2382	0.5254	0.3744	0.036*	
H7B	1.1743	0.5747	0.2906	0.036*	
C8	1.2172 (3)	0.3441 (3)	0.29856 (15)	0.0313 (5)	
H8A	1.3108	0.3534	0.2641	0.038*	
H8B	1.2427	0.2729	0.3405	0.038*	
C9	0.8982 (3)	0.4085 (3)	0.16438 (13)	0.0283 (5)	
H9A	0.8939	0.4839	0.1226	0.034*	
H9B	0.8231	0.3263	0.1520	0.034*	
C10	1.0671 (3)	0.3484 (3)	0.17388 (14)	0.0297 (5)	
H10A	1.0875	0.2650	0.1376	0.036*	
H10B	1.1469	0.4283	0.1649	0.036*	
C11	0.4878 (3)	−0.0470 (3)	0.45767 (13)	0.0287 (5)	
H11A	0.4717	−0.0173	0.5120	0.034*	
H11B	0.3821	−0.0498	0.4323	0.034*	
C12	0.5624 (3)	−0.1999 (3)	0.45463 (15)	0.0330 (5)	
C13	0.5926 (4)	−0.4222 (3)	0.3835 (2)	0.0499 (8)	
H13A	0.7093	−0.4143	0.3831	0.060*	
H13B	0.5563	−0.4686	0.3354	0.060*	
H13C	0.5589	−0.4840	0.4272	0.060*	
B1	0.9234 (3)	0.3847 (3)	0.30025 (14)	0.0236 (5)	

### Atomic displacement parameters (Å^2^)

**Table:**

	*U*^11^	*U*^22^	*U*^33^	*U*^12^	*U*^13^	*U*^23^
O1	0.0291 (8)	0.0257 (8)	0.0296 (8)	−0.0045 (7)	0.0027 (6)	−0.0029 (7)
O2	0.0318 (8)	0.0203 (7)	0.0283 (8)	0.0029 (7)	0.0023 (7)	0.0014 (6)
O3	0.0412 (10)	0.0352 (10)	0.0503 (10)	0.0057 (9)	−0.0049 (9)	−0.0132 (9)
O4	0.0875 (17)	0.0522 (12)	0.0453 (11)	0.0243 (14)	−0.0164 (12)	0.0011 (9)
N1	0.0271 (9)	0.0181 (8)	0.0295 (9)	0.0004 (8)	0.0027 (8)	0.0024 (8)
C1	0.0254 (10)	0.0195 (10)	0.0279 (10)	0.0014 (9)	0.0055 (8)	−0.0020 (9)
C2	0.0263 (10)	0.0228 (10)	0.0268 (10)	0.0030 (9)	0.0023 (9)	0.0005 (9)
C3	0.0232 (10)	0.0234 (10)	0.0318 (11)	0.0010 (9)	0.0011 (9)	−0.0018 (9)
C4	0.0289 (10)	0.0213 (10)	0.0286 (10)	0.0009 (9)	0.0075 (9)	−0.0026 (9)
C5	0.0360 (12)	0.0291 (11)	0.0236 (10)	−0.0050 (10)	0.0014 (10)	0.0013 (9)
C6	0.0282 (10)	0.0270 (11)	0.0276 (10)	−0.0031 (10)	0.0000 (9)	−0.0030 (9)
C7	0.0285 (11)	0.0286 (12)	0.0334 (12)	−0.0054 (10)	−0.0020 (9)	0.0032 (10)
C8	0.0262 (11)	0.0284 (12)	0.0393 (13)	−0.0005 (10)	−0.0021 (10)	0.0054 (10)
C9	0.0349 (12)	0.0233 (11)	0.0266 (11)	−0.0002 (10)	−0.0020 (9)	0.0003 (9)
C10	0.0347 (12)	0.0259 (11)	0.0285 (11)	0.0016 (10)	0.0062 (10)	0.0010 (9)
C11	0.0308 (11)	0.0261 (11)	0.0291 (11)	−0.0023 (9)	0.0051 (9)	−0.0008 (9)
C12	0.0345 (12)	0.0316 (12)	0.0330 (11)	−0.0013 (11)	0.0057 (10)	0.0010 (10)
C13	0.0444 (15)	0.0340 (14)	0.071 (2)	0.0026 (13)	0.0060 (15)	−0.0162 (15)
B1	0.0261 (12)	0.0188 (11)	0.0258 (11)	0.0004 (10)	0.0020 (10)	−0.0009 (9)

### Geometric parameters (Å, °)

**Table:**

O1—C7	1.417 (3)	C5—C6	1.392 (3)
O1—B1	1.449 (3)	C5—H5	0.9500
O2—C9	1.421 (3)	C6—H6	0.9500
O2—B1	1.474 (3)	C7—C8	1.525 (4)
O3—C12	1.333 (3)	C7—H7A	0.9900
O3—C13	1.455 (4)	C7—H7B	0.9900
O4—C12	1.193 (3)	C8—H8A	0.9900
N1—C10	1.488 (3)	C8—H8B	0.9900
N1—C8	1.491 (3)	C9—C10	1.523 (3)
N1—B1	1.681 (3)	C9—H9A	0.9900
N1—H1	0.9300	C9—H9B	0.9900
C1—C2	1.397 (3)	C10—H10A	0.9900
C1—C6	1.405 (3)	C10—H10B	0.9900
C1—B1	1.607 (3)	C11—C12	1.502 (3)
C2—C3	1.400 (3)	C11—H11A	0.9900
C2—H2	0.9500	C11—H11B	0.9900
C3—C4	1.391 (3)	C13—H13A	0.9800
C3—H3	0.9500	C13—H13B	0.9800
C4—C5	1.388 (3)	C13—H13C	0.9800
C4—C11	1.520 (3)		
			
C7—O1—B1	109.67 (18)	N1—C8—H8B	111.1
C9—O2—B1	110.55 (17)	C7—C8—H8B	111.1
C12—O3—C13	114.9 (2)	H8A—C8—H8B	109.1
C10—N1—C8	114.44 (19)	O2—C9—C10	105.45 (19)
C10—N1—B1	105.44 (17)	O2—C9—H9A	110.7
C8—N1—B1	103.17 (17)	C10—C9—H9A	110.7
C10—N1—H1	111.1	O2—C9—H9B	110.7
C8—N1—H1	111.1	C10—C9—H9B	110.7
B1—N1—H1	111.1	H9A—C9—H9B	108.8
C2—C1—C6	116.5 (2)	N1—C10—C9	104.23 (19)
C2—C1—B1	123.6 (2)	N1—C10—H10A	110.9
C6—C1—B1	119.9 (2)	C9—C10—H10A	110.9
C1—C2—C3	121.8 (2)	N1—C10—H10B	110.9
C1—C2—H2	119.1	C9—C10—H10B	110.9
C3—C2—H2	119.1	H10A—C10—H10B	108.9
C4—C3—C2	120.7 (2)	C12—C11—C4	110.81 (19)
C4—C3—H3	119.7	C12—C11—H11A	109.5
C2—C3—H3	119.7	C4—C11—H11A	109.5
C5—C4—C3	118.2 (2)	C12—C11—H11B	109.5
C5—C4—C11	120.3 (2)	C4—C11—H11B	109.5
C3—C4—C11	121.5 (2)	H11A—C11—H11B	108.1
C4—C5—C6	121.0 (2)	O4—C12—O3	123.2 (3)
C4—C5—H5	119.5	O4—C12—C11	124.8 (3)
C6—C5—H5	119.5	O3—C12—C11	112.0 (2)
C5—C6—C1	121.7 (2)	O3—C13—H13A	109.5
C5—C6—H6	119.1	O3—C13—H13B	109.5
C1—C6—H6	119.1	H13A—C13—H13B	109.5
O1—C7—C8	104.28 (19)	O3—C13—H13C	109.5
O1—C7—H7A	110.9	H13A—C13—H13C	109.5
C8—C7—H7A	110.9	H13B—C13—H13C	109.5
O1—C7—H7B	110.9	O1—B1—O2	112.28 (19)
C8—C7—H7B	110.9	O1—B1—C1	111.41 (18)
H7A—C7—H7B	108.9	O2—B1—C1	116.1 (2)
N1—C8—C7	103.34 (19)	O1—B1—N1	101.97 (18)
N1—C8—H8A	111.1	O2—B1—N1	100.39 (17)
C7—C8—H8A	111.1	C1—B1—N1	113.35 (18)

### Hydrogen-bond geometry (Å, °)

**Table:**

Cg3 is the centroid of the C1–C6 ring.

**Table:**

*D*—H···*A*	*D*—H	H···*A*	*D*···*A*	*D*—H···*A*
N1—H1···O2^i^	0.93	2.06	2.921 (2)	154
C10—H10B···Cg3^ii^	0.99	2.65	3.618 (2)	166

Symmetry codes: (i) −*x*+2, *y*−1/2, −*z*+1/2; (ii) −*x*+2, *y*+1/2, −*z*+1/2.
